# Seeking Solace in Gambling: The Cycle of Gambling and Intimate Partner Violence Against Women Who Gamble

**DOI:** 10.1007/s10899-022-10134-6

**Published:** 2022-06-07

**Authors:** Nerilee Hing, Lydia Mainey, Catherine O’Mullan, Elaine Nuske, Nancy Greer, Anna Thomas, Helen Breen

**Affiliations:** 1 School of Health Medical and Applied Sciences), Central Queensland University, G.07 Building 8, University Drive, 4670 Bundaberg, Queensland Australia; 2 School of Nursing, Midwifery and Social Sciences), Central Queensland University, Cairns, Queensland) Australia; 3 Faculty of Health), Southern Cross University, Coolangatta, Queensland) Australia; 4 School of Health Medical and Applied Sciences), Central Queensland University, Melbourne, Victoria) Australia; 5 Independent researcher, Melbourne, Victoria) Australia; 6 Faculty of Business, Law and Arts), Southern Cross University, Lismore, New South Wales Australia

**Keywords:** Gambling disorder, Problem gambling, Gambling harm, Domestic violence, Male partner violence, gaming machines, slots

## Abstract

This study explored women’s gambling in response to male intimate partner violence (IPV). Twenty-four women were recruited through service providers and online advertising. All women had been victimised by IPV and all experienced problems relating to the gambling on electronic gaming machines (EGMs). Thematic analysis of their in-depth interviews identified three major themes. The main *pattern of gambling and IPV* (Theme 1) was where ongoing coercive control preceded the woman’s gambling. Situational violence in response to gambling was also observed. Regardless of temporal sequence, a self-perpetuating cycle of gambling and IPV victimisation was typically apparent, with both issues escalating over time. Reflecting severe traumatic violence, *push factors from IPV that motivated the women’s gambling* (Theme 2) included physical escape, psychological escape, hope of regaining control over their lives, and gambling to cope with the legacy of abuse. *Pull factors attracting these women to gambling venues* (Theme 3) appeared to have heightened appeal to these victims of IPV. These included venues’ social, geographic and temporal accessibility, allowance for uninterrupted play on EGMs, and the addictive nature of EGMs. These push and pull factors led to these women’s prolonged and harmful gambling while exacerbating their partner’s violence. Concerted efforts are needed to assist women in this cycle of IPV and gambling, prevent violence against women, and reduce harmful gambling products and environments.

## Introduction

Research has consistently found a relationship between gambling and intimate partner violence (IPV). IPV is behaviour by a current or former intimate partner that causes physical, sexual or psychological harm, including physical aggression, sexual coercion, psychological abuse and controlling behaviours (World Health Organization, 2017). Amongst adults experiencing a gambling disorder, over one-third are perpetrators (37%) or victims (38%) of physical IPV (Dowling et al., 2016). Studies have examined physical, verbal, psychological, sexual and economic abuse amongst gamblers (e.g., Bellringer et al., 2017; Dowling et al., 2014; Hing et al., 2020a, 2021; Palmer du Preez et al., 2018; Roberts et al., 2016, 2018; Suomi et al., 2019), as well as coercive control involving use of violence to gain power and control over a partner through ongoing oppression and micro-regulation (Hing, 2020a, Johnson 2008; Stark, 2009).

Research has identified three main patterns linking IPV and gambling. One is where the person who gambles perpetrates IPV. This violence may arise from the gambler’s anger and frustration about gambling losses, expressed as an immediate aggressive response to financial stress and crisis (Afifi et al., 2010; Korman et al., 2008; Muelleman et al., 2002; Suomi et al., 2019). A second pattern is where the person who gambles is a victim of a partner’s abuse which may arise from loss of trust, accumulated anger, and conflict over stresses caused by the victim’s gambling (Korman et al., 2008; Suomi et al., 2013). These two patterns have been described as situational couple violence (Johnson, 2008) triggered by the immediate effects of gambling (Suomi et al., 2019). However, these violent incidents can also occur in contexts of coercive control and non-gambling related violence; that is, gambling can intensify violence already occurring in abusive relationships (Hing et al., 2020a). A third pattern is where victims gamble to cope with past or current IPV (Afifi et al., 2010; Hing et al., 2020a; Suomi et al., 2019). In their study of 212 clients of gambling help services, Suomi et al., (2019) observed this pattern only amongst women and suggested their abuse was likely to be severe and traumatic and linked to a pattern of coercive and controlling behaviours by their partner. Suomi et al., (2019) indicated the need for further research to better understand this pattern of IPV and gambling amongst women. The current study helps to address this need by focusing on women victims of IPV who use gambling to escape from or cope with IPV.

IPV has immediate and lasting impacts on women which may increase their likelihood of gambling. Women victims are at risk of mental health problems, including depression, anxiety and post-traumatic stress disorder (PTSD) (Dillon et al., 2013; Lagdon et al., 2014). Women experiencing coercive control may live in a state of hypervigilance and fear (Felson & Outlaw, 2007; Gondolf & Heckert, 2003). Women victims also have an elevated risk of physical health problems (Dillon et al., 2013; Laskey et al., 2019), and substance use disorders (Cafferky et al., 2018; Devries et al., 2014). The self-stigma of IPV victimisation and a partner’s controlling behaviours can lead to social isolation and loneliness (Laskey et al., 2019). IPV often has negative financial consequences, including economic abuse (King et al., 2017; Ulmestig & Eriksson, 2017). These impacts of IPV on women may motivate their use of gambling to cope with these difficulties, and to provide a direct escape from the violence itself.

Women’s gambling has been widely researched, although not in the context of IPV. Of note is women’s preference for playing electronic gaming machines (EGMs), often for emotional relief from stressful life circumstances, boredom, loneliness, anxiety and depression (Grant & Kim, 2002; Hing et al., 2017; Thomas et al., 2011a). While some men also gamble for emotional escape, women are more likely to be seeking respite from violent partners, stressful relationships, caring responsibilities, social isolation, emotional distress, mood disorders and life worries (Saugeres et al., 2012; Thomas & Moore, 2003). Gambling may also be used to cope with violence-induced trauma, reflected in higher rates of abuse amongst individuals with a gambling disorder (Afifi et al., 2010; Andronicos et al., 2015; Kausch et al., 2006). EGM play assists avoidant-based coping because it facilitates dissociation and trance-like absorption. Players describe this state as “the zone” where reality is suspended, life’s problems lose importance, and players can numb pain and worries (Livingstone, 2005; Schüll, 2012). The goal in “the zone” is to sustain play to extend time-out from a difficult reality (Schüll, 2002). However, while EGM play may help women to cope with the impacts of IPV victimisation, it can lead to dependence and harm, and exacerbate the risk of further violence. Women have described gambling to cope with abusive intimate relationships, and sometimes experiencing further violence after subsequent gambling losses (Centre for Innovative Justice, 2017; Saugeres et al., 2014).

Women also find EGMs attractive because they are highly accessible. In Australia, nearly all suburbs have several hotels and clubs with EGMs which open for up to 18 h every day (Hing et al., 2017). They are often the only venues open late at night. EGM venues are also socially accessible for women, where they feel socially accepted when playing EGMs and can expect to be left alone (Thomas et al., 2009, 2011b). Security personnel, employees and other patrons enhance women’s sense of safety. Venue staff are trained to be welcoming but non-intrusive and to not question a patron’s gambling (Hing et al., 2020b; Rintoul et al., 2017). In Victoria Australia, the Royal Commission into Family Violence (State of Victoria, 2016) found that women sometimes escape to gambling venues because they are the only safe, welcoming refuge to avoid a partner’s violence; yet cautioned that venues are not safe if this leads to harmful gambling and the risk of more violence.

While research has found that some women gamble to cope with IPV Centre for Innovative Justice, 2017; Saugeres et al., 2012; State of Victoria, 2016; Suomi et al., 2019), little is known about the interaction of IPV and gambling in this context and the experiences of affected women. The current study aimed to explore women’s gambling in response to IPV victimisation by drawing on their lived experience. Specifically, it explored the following research questions: (1) What is the nature of the relationship between gambling and IPV when women gamble in response to IPV victimisation? (2) How does IPV victimisation contribute to women’s gambling? and (3) Why are gambling venues attractive as “safe spaces” for women victims of IPV?

## Methods

### Participant recruitment

Given the paucity of related research, a qualitative study was undertaken to explore the research questions. After obtaining ethics approval from the lead author’s institution (no. 20,852), participants were recruited through service providers working in family violence, relationship counselling, gambling help, financial counselling and multicultural agencies, as well as through paid advertising on GoogleAds and Gumtree. Potential participants were directed to a dedicated website which contained an information sheet that explained the study was:

… seeking to interview women located in Australia who have engaged with a domestic violence, gambling or allied support service and have had any of the following experiences:

- You have had problems with gambling, and a current or former male partner used controlling or violent behaviours towards you.
- You have used gambling or gambling venues to cope with or escape from controlling or violent behaviours towards you from a current or former male partner.

The information sheet also explained provisions for confidentiality and anonymity, voluntary participation, contact details for help services, an informed consent preamble, and payment of a $40 shopping voucher as compensation. Potential participants could register on the website or by contacting a nominated researcher. This researcher then contacted the woman to check she met the eligibility criteria described above, and if so, to arrange an interview time.

Twenty-four women participated in interviews, with nearly half (11) aged below 40 years and the rest (13) aged 40 years or over (Table 1). Most (19) resided in metropolitan areas. The sample included women from five Australian jurisdictions, mainly from the most populous eastern states: Queensland (9), New South Wales (7) and Victoria (4). All 24 women reported EGMs (also called poker machines and pokies in Australia) to be their most harmful form of gambling.

**Table 1:** Key characteristics of the participants

No.	Participant ID	Age group (years)	State	Location type
1	WLE003	60–69	Victoria	Regional
2	WLE010	40–49	Queensland	Metropolitan
3	WLE012	20–29	Queensland	Metropolitan
4	WLE017	50–59	Queensland	Metropolitan
5	WLE019	60–69	Queensland	Regional
6	WLE024	50–59	Victoria	Metropolitan
7	WLE031	40–49	Queensland	Metropolitan
8	WLE033	30–39	Queensland	Metropolitan
9	WLE034	30–39	Victoria	Metropolitan
10	WLE038	60–69	Queensland	Metropolitan
11	WLE045	50–59	South Australia	Metropolitan
12	WLE046	20–29	Northern Territory	Metropolitan
13	WLE048	30–39	South Australia	Metropolitan
14	WLE049	30–39	New South Wales	Metropolitan
15	WLE050	30–39	South Australia	Metropolitan
16	WLE055	30–39	New South Wales	Metropolitan
17	WLE057	40–49	New South Wales	Metropolitan
18	WLE061	60–69	Queensland	Metropolitan
19	WLE062	20–29	New South Wales	Metropolitan
20	WLE063	30–39	New South Wales	Metropolitan
21	WLE067	50–59	New South Wales	Regional
22	WLE069	30–39	New South Wales	Regional
23	WLE070	60–69	Queensland	Regional
24	WLE086	40–49	Victoria	Metropolitan

### Interview procedure

Four female researchers conducted the interviews. The interviewer confirmed the participant’s informed consent and checked they were in a safe location. To ease the women into the interview, they were first asked why they were participating. They were then asked to share their story of how IPV and gambling had impacted their life. The interviewer suggested:

You might start from when problems first started occurring and tell me how things developed over time. I’m particularly interested in the role that gambling played in the abuse you have experienced, or how the abuse might have affected your gambling.

The interviews were conducted in a conversational style with their direction largely guided by participants, although the interviewer would prompt for clarification, details or examples if needed. The interviewers had pre-prepared topic areas (e.g., types of gambling, nature of the abuse, services used), and asked about areas that had not been covered during the woman’s narrative. All women said they were participating to help women in similar situations and were therefore prepared to give detailed accounts. The interviews lasted 50–90 min, were digitally recorded with permission and professionally transcribed. Participants were given the opportunity to check and revise their transcript.

### Data analysis

Thematic analysis of the interviews was conducted for the current study and adhered to the steps articulated by Braun and Clark (2006). After familiarisation with the data by reading the transcripts multiple times, two of the interviewers/researchers proceeded line by line through each transcript to generate initial codes. This open coding was an inclusive and systematic process to identify initial features of potential relevance to the research questions. In working through all interview transcripts, this inductive iterative process used the constant comparative method to add new codes, modify existing codes, and recode data as appropriate. In an iterative process of review and revision, five researchers then reviewed the coded data to identify similarity and overlap, and clustered codes with unifying features to capture meaningful themes and sub-themes in the data. After finalising the themes and sub-themes, three researchers then reviewed the interview transcripts and helped saturate the themes with further evidence from the data, including participant quotes.

## Findings

Three main themes and several sub-themes were identified (Table 2) and are discussed below.

**Table 2:** Themes and sub-themes related to women’s gambling in response to IPV

Themes	Sub-themes
Patterns of gambling and IPV	Ongoing coercive control prior to gambling Situational violence in response to gambling
Push factors from the IPV that motivated women’s gambling	Physical escape from IPV Psychological escape from IPV Hope of regaining control over their lives Cope with loneliness and trauma after leaving the relationship
Pull factors that attracted women to gambling venues	Social accessibility of EGM venues Venues do not interrupt EGM play Geographic and temporal accessibility of EGM venues The addictive nature of EGMs

### Patterns of gambling and IPV

Two main patterns of gambling and IPV were identified.

#### Ongoing coercive control prior to gambling

The most common temporal sequence amongst this cohort of women was being subjected to IPV, often over many years, prior to their commencement or regular engagement in gambling. The violence experienced was severe and included being denigrated, gaslighted, physically assaulted, choked, stalked, sexually assaulted, and financially exploited. Consistent with a pattern of coercive control, all these women reported that their partner used monitoring, subordination, threats and violence to restrict their liberties and wield power over their activities, relationships and everyday behaviours. Most women explained that their partner’s violence was ongoing and characterised by self-centred, controlling and coercive behaviours: “narcissistic … very controlling and very blaming … always my fault … he likes to control what I can and can’t do, like I wasn’t allowed to do anything” (WLE050). Mental health problems, alcohol and drugs were often said to further exacerbate these men’s violence.

These women sought to cope through seeking physical safety in gambling venues or emotional respite through playing EGMs: “any time he’s abusive towards me, it seems to be my go to” (WLE057). They found that their gambling soon escalated. For example, one woman who was “terrified” by her partner’s violence and death threats explained “when the abuse started that’s when I used it as an escape, and that’s when the real addiction took over, like, the lack of control” (WLE086). Women seeking refuge from violence by gambling in EGM venues faced inevitable gambling losses which exacerbated the ongoing violence from their partner: “He said ‘did you lose it all?’… and he just gave me a hell of a hiding” (WLE017). However, violence was an ongoing feature in these relationships and not necessarily perpetrated only in direct response to the woman’s gambling.

#### Situational violence in response to gambling

A far less common pattern in this cohort was where the woman’s gambling preceded her victimisation. These women reported various contributors to their uptake of gambling, including mental health issues, loneliness, boredom, relationship dissatisfaction, financial stress, and irrational beliefs about gambling. However, most of these women reported that their partner’s abuse increased after they commenced gambling, with his subsequent behaviour characterised by ongoing denigration, insults, blame and angry outbursts which sometimes involved physical violence. This woman recounted how her partner had come to the gambling venue to abuse her:

He came in and stood behind me playing the poker machine, and he said something out really loud … like, “Fucking spending - blowing all our money, fucking good on ya” … and stormed out, and people all around me looked … not long later, I heard them announce over the loudspeaker, my name, “Please come to reception” … he’d left a message saying – for me to go home immediately. (WLE031)

While the abuse was initially situationally-provoked and commenced in response to the woman’s gambling, it sometimes became an ongoing pattern of behaviour. Women increasingly sought refuge in gambling venues as the abuse escalated, which in turn exacerbated their gambling. For example, this participant explained that her partner’s abuse was initially in response to her gambling, but he then used her gambling as an excuse for continued abuse. She gambled more to escape his behaviour:

The main reason we started arguing was because I was gambling ... I know that there were several factors why it [the violence] happened, but he solely blames it on the gambling … Once we were full on fighting, no matter what the reason, I started using the gambling as an outlet to get away from him, as an escape. (WLE010)

### Push factors from the IPV that motivated the women’s gambling

Several “push factors” arising from the IPV were said to motivate the women’s gambling.

#### Physical escape from IPV

Most women spoke about visiting EGM venues to physically escape from their partner’s abuse because it was “safer than being at home” (WLE045). One woman whose partner continually degraded her, saying she was fat, horrible and no one else would want her, and who threw unwanted meals she had prepared at her, explained: “I went to the pokies because I could escape what he was saying to me” (WLE048). Another woman, whose partner abused her if the housework did not meet his standards, controlled her social interactions, and made her pay most household expenses, noted “it was my escape to get away from having to deal with all that” (WLE012).

Women particularly sought refuge in EGM gambling venues when their partner’s violence was escalating. This coping strategy was a major contributor to these women’s gambling. For example, two recalled how their need to escape violence led to their dependence on EGMs as they spent long hours in venues:

I was actually using the pokies as an escape from the domestic violence. So, when the violence and the emotional abuse would erupt, I would leave the house, because I had no friends or family around me. So, I would actually go the pokies and that’s where I would stay. I was never coming home. I didn’t want to be at home. (WLE086)

He did drink a lot. So, I would run away … to the boat club. I wouldn’t leave until it was closed and maybe he was home and hopefully asleep. Then, I would creep in … [If] I’d wake him up, I didn’t know what I was in for. (WLE017)

EGM venues were frequently the only safe space for these women because they were isolated from friends and family due to their partner’s oppression or their shame about the IPV. One explained “It was either go to the pokies or sit in a car park somewhere in my car, because I had nowhere else to go or no one to talk to” (WLE086). Another participant, whose “bizarrely jealous” and “controlling” partner tormented her through gaslighting and taunted her about his infidelity, recounted how he was barred from a venue because of his behaviour: “so I was able to go there as a safe place, because he wasn’t allowed in” (WLE010). Over time, she had nine domestic violence orders against him due to his menacing and violent behaviours. However, women also noted that their physical safety in venues was temporary and elevated the risk of further abuse when they went home: “That was my safe haven … until you left … [and then] it was worse” (WLE045).

#### Psychological escape from IPV

The violence severely eroded all women’s mental health and motivated their gambling as a psychological escape from stress, anxiety and trauma. One woman who had been hospitalised several times by her partner explained “I found myself going to the pokies to pass the time and just to help with my anxiety” (WLE017). She described the dissociation and distraction that EGMs provided:

It was like a relief to watch the things spin around and you’d think about that, instead of thinking about anything else … the anxiety just quietly dissipates … oh I got two little Indian fellas and I’ve won something, and that was refreshing from everything else that was going on. It was relief and a distraction. (WLE017)

Another woman, whose partner physically and verbally abused her, locked her in his room when he went to work, and took her to the bush and threatened to kill her, recounted how she found “peace and escape” in her gambling, that “it can be so therapeutic, it’s amazing … the rhythmic thing … it’s meditative” (WLE055). She continued:

The reward system in my brain, it’s just drained out … I can still sit and worry about crap. Whereas when I’m on the machines, it’s a lot easier to be fixated on the patterns and the mathematics of it. (WLE055)

Several women were diagnosed with PTSD due to their abuse. One woman described the violence from her drug-dependent partner. This included ongoing physical assault, being punched in the face, and having a rope put around her neck, along with violent retaliation if she questioned his behaviour. She described “this constant power struggle” where “he was one of those guys that had to know where I was every minute of the day”. She referred to the comfort she sought in gambling venues to try to cope with her ongoing trauma:

It’s almost like when something [violence] happens I get all these flashbacks to all the other incidents and then I seem to find myself in the comfort of a poker machine room. (WLE057)

#### Hope of regaining control over their lives

Some women gambled because it gave them hope for a better life, some control, and a potential financial ticket out of their situation. One participant spoke about her abusive upbringing where “all my family have hit me or abused me” such that she became “desensitised” to violence. She went from one violent relationship to another “very cold, violent, bitter and stalkerish” man who had tried to choke her, slammed her face into the road, dragged her down a 30-metre driveway, and sexually assaulted her. Playing EGMs provided hope of being able to escape and gain some control over her life:

There’s an element of hope. And even if it lets you down, it’s no different to everything else that lets you down; but that’s still got the odds with it. And also, yeah, the rhythmic escape, the sense of purpose. Like, I’m practically contributing to my life, because I could win, and I could drastically change my situation. (WLE055)

The hope of a financial windfall so she could leave also motivated the following woman’s gambling. After enduring her partner’s ongoing cruelty, violence and coercive control, she was later diagnosed with PTSD:

The situation really escalated after our son was born and he really tried to control everything about what I was doing and parenting and just everything. At that point I felt trapped … in the back of my mind thinking, my god, if I got a big windfall I’m just going to leave. (WLE057)

One woman gambled in the hope of winning because her partner was less likely to be abusive if she gave him money. Gambling became a way to placate him and protect herself from further abuse: “For me, it became, ‘alright, I’ve had a win, it will be fine’” (WLE017). Some women trying to regain control of their situation gambled in secret, to have something for themselves. This woman described how “All my sense of self was slowly bled out” by her controlling partner who restricted her movements and ridiculed any outside interests she had. She reported gambling to try to recapture her independence and identity:

It was also sort of a secret alone thing *…* Maybe somewhere in my mind I just felt entirely consumed by this other person. Maybe it was a way of regaining my sense of self or my own identity. (WLE062)

However, trying to regain independence from a coercive and controlling partner risked violent retaliation, as this participant shared: “I started to try and regain my power. But that only made things worse … He got angrier and angrier … before he beat the shit out of me” (WLE057).

#### Cope with loneliness and trauma after leaving the relationship

After escaping violent relationships, women were often left with a legacy of bitter memories, loneliness, and poor mental health. Some women commenced gambling after the relationship ended or continued gambling to cope with the lasting effects of their victimisation: “For a good couple of years I was very raw and very untrusting, almost agoraphobic. But I would find solace in gambling” (WLE017). Many women remained unpartnered into middle and older age. They found that playing EGMs helped to distract them from life’s problems, loneliness, boredom, and going home to an empty house. Two women explained gambling to cope with their struggles to find a meaningful role in later life:

When I’m there [at the EGM], it’s just a dead zone, it’s a vacant zone … I can just go and have a fling and just forget everything … before I go home to an empty house … you think, god, you’ve had this whole life, doing all this stuff, and here you are on your own, what’s wrong with you? (WLE061)

The worst thing for me was that … as my kids grew and they left home … I’d come home from work and there’d be nothing, you know? … I had nothing to do. (WLE024)

Gambling was an escape mechanism for these women to cope with the legacy of their abuse. Gambling provided a psychological escape from their continuing distress and trauma. EGM venues also provided a physical escape from their empty, lonely house after the relationship ended, in contrast to using EGM venues to physically escape from the violent, conflict-ridden house when in the relationship.

### Pull factors that attracted women to gambling venues

Several “pull factors” were said to particularly attract the women to gambling venues.

#### Social accessibility of EGM venues

Several aspects of EGM venues enhanced their social accessibility for women. One was feeling that it was socially acceptable to patronise venues and play EGMs on their own without attracting unwanted attention. Participants felt there were few alternative recreational activities they could engage in alone in women-friendly environments: “It’s the one thing I can do when I get out by myself” (WLE31).

Several women enjoyed the hospitality in these venues: “they handed you food – sometimes, you got points and you got drinks … they make it so easy to be appealing” (WLE010). Some participants noted that the welcoming, comfortable facilities were a stark contrast to their home environment. One recalled the first time she went to a venue to delay going home to her abusive husband after work:

There were all these people, and free snacks, and lights, and noise and music; and I thought, “Oh, this is nice” … At the time, it wasn’t gambling, it was just an excuse to spend time in this lovely environment, which was so welcoming, and unlike anything else I had to go to. (WLE019)

Many women had become socially isolated, especially where male partners controlled and restricted their relationships and activities. The social environment in gambling venues enabled these women to be around other people and escape their isolation:

It’s a friendly environment, there’s other people there and people at the bar are quite friendly and I need to be around people … I have limited friends these days and it’s like just … being around everyone I suppose. (WLE045)

Many women spoke about their positive interactions with venue staff who were friendly, paid them attention and enquired after them, which was unlike their situation at home:

They’d say, “How’s your day going?” … Otherwise, nobody would have talked to you all day … Just loved it. And I really felt that you had to be either drinking or playing the pokies to be there. (WLE019)

Crucially, these interactions were superficial and non-threatening, allowing women to maintain privacy and keep their domestic problems hidden. However, while EGM venues may appear to be “safe spaces” that provide a nurturing environment, they are unsafe for vulnerable women who then develop a gambling problem.

#### Venues do not interrupt EGM play

In contrast to the above quotes demonstrating the desire for social interaction, many women reported that they wanted to be left alone to play EGMs without interruption or judgment, to zone out and numb the stress and trauma of their abuse: “I don’t like to talk to anyone while I’m at the club … I like to just focus on my screen” (WLE069). Two other women explained how they chose to keep to themselves in the venue and were able to do so:

I always sat by myself … I never got comfortable talking to people, particularly if I had makeup on covering a black eye, which happened more often than not. (WLE017)

I don’t want to talk to other people, especially men there … I just can pass away time … You don’t even think anything. Except “oh my god, I’ve run out of money. Oh, maybe I could just get another $50 out.” (WLE024)

Gambling venues facilitated this uninterrupted play and made no attempt to intervene with the participants’ gambling: “they just tend to turn a blind eye” (WLE031). Signs of harmful gambling would have been very obvious in some women, such as one participant who had lost between $400,000 and $500,000 and would leave the venue in tears (WLE034), and another who withdrew money from the venue’s automatic teller machine seven times in one session (WLE031). However, at no time did venue staff ask any of the participants about their gambling or need for support.

#### Geographic and temporal accessibility of EGM venues

Venues located close to home increased their attractiveness as a refuge from violence. Most women patronised just one or a few local venues. This convenient access when seeking a physical or psychological escape from their partner’s abuse contributed to their gambling. To remove this convenience, one woman moved cities to distance herself from EGMs. However, access to EGMs in her new location hindered these attempts:

I thought I need to be in a job where I’m absolutely away from these things, and of course I got to Darwin then blow me down, they’ve got an EGM venue down the road. (WLE003)

Several women talked about being able to access gambling venues at nearly any time due to their long opening hours. While this allowed women to seek safety from their partner’s abuse, particularly late at night when other public facilities are closed, it also compounded the frequency and extent of their gambling: “You can just turn up at any time, like for instance in the middle of the night” (WLE049).

#### The addictive nature of EGMs

While most women had commenced gambling in response to their abuse, their subsequent dependence on EGMs and their continued use of venues as a refuge from violence strengthened their attraction to gambling: “it became habitual. You start chasing the money, I guess. So, I started looking for excuses to go as well.” (WLE017). Several women spoke about the addictive nature of EGMs that “give that hypnotic effect to people, to suck them in … Gambling would have to be the top addiction … the hardest of them all to conquer” (WLE033). Another commented:

I’m absolutely convinced that these poker machines will, by the very nature of their design, they will addict to any vulnerable human brain, and that human brain can be quite a normal human being and a normal brain, without any preconceived type of, well, psychological problems or anything like that. (WE003)

One participant highlighted the strength of her attraction to EGMs with the following anecdote:

I drove past the local pub, and it had a big flashing sign out, “Pokies, pokies”, and I just could not help myself … the urge was just eating me … I went to Gamblers Anonymous and stopped at the pokies on the way home. (WLE031)

## Discussion

This study has explored women’s gambling in response to IPV victimisation and in relation to three research questions. The first question focused on the nature of the relationship between these women’s gambling and their victimisation. Most women had been subjected to years of ongoing coercive control, accompanied by regular incidents of violence by their partner before they commenced or intensified their gambling. This pattern aligns with one observed in previous research where a victim gambles to cope with IPV (Afifi et al., 2010; Suomi et al., 2019). While this abuse was not necessarily perpetrated in direct response to these women’s gambling, their gambling gave already abusive perpetrators a further excuse to use violence against them. This finding supports the conceptualisation of gambling as a reinforcing contributor to violence against women that increases its frequency and severity when other drivers of violence are present in the relationship (Our Watch et al., 2015). Drivers of violence against women are widely recognised to be gender-based and founded in unequal power relationships and gender stereotypes, where abusive male partners assume a right to control the woman and prioritise their needs above hers (World Health Organization, 2017). Women in this study who were subjected to coercive control characterised their partner as self-centred, controlling and domineering, reflecting an assumed hierarchy in the relationship and a perceived entitlement to use violence, coercion and control to subordinate a female partner. Gambling often became an additional pretext to exercise this abuse, along with other reinforcing factors such as alcohol and drug use by the perpetrator.

A minority of women first experienced IPV after they commenced gambling. Their initial experiences of violence might be considered situational couple violence (Johnson, 2008), being an immediate response to the woman’s gambling (Korman et al., 2008; Suomi et al., 2013, 2019). However, these women’s subsequent victimisation was not always limited to violent incidents in direct response to their gambling, although these continued to occur. Instead, some partners’ abuse came to permeate the relationship and manifested as ongoing denigration, disrespect and violence. Women reported intensifying their gambling over time in response to this subsequent and continued abuse. Overall, regardless of whether the IPV preceded the gambling or vice versa, a self-perpetuating cycle of gambling and IPV victimisation developed for the women in this study. Rather than gambling leading to IPV *or* IPV leading to gambling, the nature of the relationship between these women’s gambling and their IPV victimisation tended to be cyclical, self-reinforcing, and characterised by an escalation of both issues over time.

The second research question focused on how IPV victimisation contributes to a woman’s gambling. Our findings are consistent with Suomi et al.’s observation (2019) that women who gamble to cope with IPV have typically experienced severe traumatic violence. Most women reported a pattern of violence referred to as intimate terrorism (Johnson, 2008). This involves ongoing tactics including economic control, emotional abuse, isolation, blaming, intimidation, coercion and threats, enforced through an assertion of male privilege and the threat or reality of physical and sexual violence (Pence et al., 1993). This coercive control is motivated by the desire to exercise power and control over the woman, and is instrumental and sustained (Johnson, 2006; Kimmel 2002; Stark, 2009; Tanha et al., 2010). Women in the current study had typically experienced years of intimate terrorism, reflected in multiple DVOs against many partners. As also observed in previous investigations (Centre for Innovative Justice, 2017; State of Victoria, 2016), fear of physical violence from their partner drove women to seek safety in gambling venues, particularly when arguments erupted, he had been using alcohol or drugs, or the women sensed that tension was building.

All women played EGMs as a psychological escape from the impacts of IPV. While women’s use of EGMs for avoidant-based coping is well documented (Saugeres et al., 2012; Thomas et al., 2011a), findings from this study indicate that the psychological effects of IPV on women’s mental health are a powerful driver of harmful EGM play. Violence-induced trauma, along with the stress, anxiety and social isolation of being in a violent relationship, drove these women to seek solace in sustained EGM play where their fears and worries could be temporarily suspended (Livingstone, 2005; Schüll, 2012). Some women commenced or continued playing EGMs after escaping from the relationship to cope with the legacy of PTSD, loneliness and boredom. Women also played EGMs in the hope of a large win so they could leave the relationship and regain their control and self-identity. Lack of financial independence traps women in violent relationships (Adams et al., 2008; Corrie et al., 2013; Weaver et al., 2009), and gambling provides one of the few sources of hope for women in these situations. However, this is a false hope, given that gambling and chasing losses on EGMs rarely result in financial gain and in fact compounded these women’s problems.

The third research question focused on why gambling venues are attractive as “safe spaces” for women victims of IPV. Aspects of venues that the women found attractive were consistent with those previously identified by women who gamble on EGMs. These include being a socially accessible environment where women can feel welcome, comfortable and safe, that allow for both social interaction and solitude, and are close to home (Hing et al., 2017; Thomas et al., 2009, 2011b). The attractiveness of these features may be amplified for women victims of IPV. Consistent with previous research (Johnson, 2008; Laskey et al., 2019), many women were socially isolated due their partner’s restrictiveness, along with the women’s shame about their IPV victimisation and gambling. Gambling venues provided one of the only recreational outlets where these women could avoid feeling judged and keep their domestic problems hidden, while enjoying the hospitality, social environment, and pleasant but superficial interactions with staff. The venues’ welcoming atmosphere was in stark contrast to these women’s home environments which were characterised by tension, fear and abuse. Unlike at home, they could feel safe due to the presence of other people and could find some peace within “the zone” of EGM play. Importantly, venues were conveniently located and open for long hours, meaning the women could seek an escape if violence was brewing at home. Overall, the features of gambling venues that women find attractive appeared to have added appeal to these victims of IPV. However, while these “safe spaces” helped to meet the needs of these women for physical safety and psychological nurturing, they proved to be very unsafe spaces that exacerbated their gambling and the cycle of IPV victimisation.

Like all research, this study has limitations. The sample was small and self-selecting, with the study design prioritising rich insights over generalisability. The small sample did not enable analysis by sub-groups, such as women with different cultural, socio-economic or demographic characteristics, who may have distinctive experiences of IPV and gambling. The sample was also limited to women who had sought professional help for their gambling or IPV; including women who had not sought help may have yielded additional insights. Further research is needed to ascertain the generalisability of the findings in larger samples and to measure the prevalence of gambling to cope with or escape from IPV victimisation.

## Conclusions

This study has provided an in-depth exploration of the lived experience of women who gamble in response to IPV victimisation. The combination of push factors from the IPV and pull factors from gambling venues resulted in the women’s prolonged and harmful use of EGMs, while exacerbating the frequency and severity of their partner’s violence. These findings implicate several measures that could help women caught in this cycle of violence. Alternative safe spaces are needed for women to escape from situations of violence which do not pose the risk of gambling harm. Gambling venues need more proactive measures to identify and support patrons showing signs of harmful gambling. They could also be alert to signs of victimisation amongst their female patrons and provide information and support to seek help. Gambling help services need to screen for IPV, while IPV services should screen for gambling, and be able to refer women to appropriate sources of help. Both IPV and gambling cause serious harm to women and are even more dangerous in combination. Concerted efforts are needed to assist women in this cycle of IPV and gambling, prevent violence against women, and reduce harmful gambling products and environments.

## Data Availability

Data not available due to confidentiality reasons.
